# Humor experience facilitates ongoing cognitive tasks: Evidence from pun comprehension

**DOI:** 10.3389/fpsyg.2023.1127275

**Published:** 2023-01-27

**Authors:** Wei Zheng, Xiaolu Wang

**Affiliations:** ^1^School of Foreign Languages, China Three Gorges University, Yichang, China; ^2^School of Foreign Languages, Hangzhou City University, Hangzhou, China; ^3^School of International Studies, Zhejiang University, Hangzhou, China; ^4^School of Humanities and Communication Arts, Western Sydney University, Kingswood, NSW, Australia

**Keywords:** humor experience, homophone puns, facilitative effect, cognitive task, embodied cognition

## Abstract

Empirical findings on embodied cognition have shown that bodily states (e.g., bodily postures and affective states) can influence how people appreciate humor. A case in point is that participants were reported to read pleasant sentences faster than the unpleasant controls when their muscles responsible for smiling were activated. However, little research has examined whether the feeling of amusement derived from humor processing like pun comprehension can exert a backward influence on ongoing cognitive tasks. In the present study, the participants’ eye movements were tracked while they rated the comprehensibility of humorous sentences (homophone puns) and two types of unfunny control sentences (congruent and incongruent). Fixation measures showed an advantage in the critical homophone region for the congruent controls relative to the homophone puns; however, this pattern was reversed in terms of total sentence reading time. In addition, the humor rating scores acquired after the eye-tracking experiment were found negatively correlated to the overall sentence reading time, suggesting that the greater amusement the participant experienced the faster they would finish the rating task. Taken together, the current results indicate that the positive affect derived from humor can in turn provide immediate feedback to the cognitive system, which enhances text comprehension. As a result, the current finding provides more empirical evidence for the exploration of the interaction between the body and cognition.

## Introduction

Humor is a crucial component of human social interaction and cognitive functioning (Vrticka et al., 2013). Although nearly all of us can easily detect humor when we experience it, we may still find it quite challenging to give a precise definition of humor. Indeed, the notion of humor is quite general and can vary from person to person. In this paper, we adopt the definition given by Martin and Ford (2018) that humor is a comprehensive term consisting of a verbal or non-verbal stimulus that people perceive as funny, the mental processes of generating or perceiving such an amusing stimulus, and as well as the emotional response of mirth. In particular, we focus on verbal humor, namely, humor expressed through language, rather than non-verbal ones such as comedy performances.

Previous research has shown that the positive emotion associated with humor can lead to cognitive benefits, especially in creativity and memory. Indeed, researchers have long noted the positive correlation between humor and creativity (Koestler, 1964), and have postulated at least two hypotheses on why humor may be beneficial for creativity. The Flexible Thought Hypothesis argues that the flexible cognitive processes involved in understanding humor can boost the divergent thinking necessary for creative tasks (Belanger et al., 1998). The Positive Emotion Hypothesis emphasizes that the positive emotion we experience in humor can reduce tension and anxiety, which in turn can promote our thinking and enhance our ability to integrate divergent materials (Isen et al., 1987). In addition, convincing evidence has also accumulated from empirical studies supporting the idea that humor can enhance memory. Specifically, researchers have found that humorous materials are recalled better when presented in educational settings (Ziv, 1988), advertising settings (Krishnan and Chakravarti, 2003), or research settings (Chambers and Payne, 2014).

Despite such cognitive benefits from a relatively long-term point of view, recent humor theories, especially cognitive-perceptual ones, predict a processing disadvantage for online humor comprehension. The Incongruity-Resolution Model, for example, argues that understanding a humorous text involves two separate stages (Suls, 1972; Attardo and Raskin, 1991). When reading humorous texts, such as a joke, the reader actively forms a discourse representation based on the input from the set-up sentences. However, this mental representation is then disconfirmed by the punchline, resulting in the detection of incongruity (the first stage). To get the joke, the reader has to search for the other plausible but less likely interpretation hinted by the punchline and resolve the semantic dilemma (the second stage). Take the following joke, for example.

Son: *Daddy, what is an alcoholic?*

Father: *Do you see those four trees, son? An alcoholic would see eight trees.*

Son: *Um, Dad, there are only two trees*.

Before reading the last sentence of the dialog, one is likely to assume that it is a regular conversation about a sober father teaching his son something. However, the disparity between the number of trees reported by the father and son (four vs. two) leads to the incongruity, which triggers the reader to work out another interpretation to incorporate the new information. Once the reader realizes that it is not a sober but drunken father who is talking to his son, then the incongruity is resolved and the humor occurs. According to this theory, the incongruity-resolution process in a joke costs more cognitive resources than a straight ending (e.g., Son: “*Um, Dad, I understand it now*.”), hence prolonged reading times for the punchline (See also the standard pragmatic view, Grice, 1975).

The Space Structuring Model also predicts a disadvantage for the processing of humorous materials (Coulson et al., 2006). Adopting the framework of cognitive linguistic theories, such as Mental Space Theory, Conceptual Blending, and Frame Semantics, this theory claims that background and contextual information plays an active and vital role in the meaning-construction process rather than just helping to disambiguate the meaning of ambiguous words. In the case of humor comprehension, the Space Structuring Model proposes “frame shifting” as the central process for getting the humor, in which a new frame is retrieved from the long-term memory when the incoming information is inconsistent with the currently-activated frame (Coulson, 2015). Due to this extra cognitive process, it also predicts extra processing efforts for reading humorous materials.

The predicted disadvantage of processing humorous materials has been demonstrated in several empirical studies. Coulson and Kutas (1998) employed the self-paced reading paradigm to investigate the processes of joke comprehension. In their experiment, the authors used one-line jokes and manipulated the relationship between preceding contexts and the sentence-final words so that the final words either trigger a frame-shifting process or remain consistent with the currently-activated frame (e.g., *I asked the woman at the party if she remembered me from last year and she said she never forgets a dress/face*). It was shown that the participants spent longer reading times on the final words in the humorous condition (e.g., *dress*) than in the non-funny control condition (e.g., *face*). Since the final words in both conditions were matched in cloze probability, these results were interpreted as reflecting the additional cognitive cost for the frame-shifting process. Coulson et al. (2006) replicated this processing disadvantage for jokes in an eye-tracking experiment using the same materials. In addition to longer reading times, the participants were also found to regress more into the previous context from the sentence-terminal position, indicating the readers needed to re-evaluate the relationship between the punchline and the discourse representation built upon the sentential context. In a recent ERP study, Mayerhofer and Schacht (2015) also reported increased reading times for the final words in the so-called garden-path jokes compared to those in sentences with coherent endings. Take one of their jokes for example. *Mummy, I just turned 14. May I please, finally, be allowed to wear a bra and makeup?* –*No, you are not. Eat up your soup, my son!* In this joke, the initial interpretation is disconfirmed by *my son* and readers have to reanalyze the speakers’ relationship in order to form a new coherent representation. Additionally, joke endings also elicited enhanced N400 amplitudes, indicating greater integration difficulties (Kutas and Federmeier, 2011).

In contrast to the above-mentioned findings, results from other studies have suggested that humor facilitates online cognitive processing. Mitchell et al. (2010), for example, reported a humor facilitation effect when the participants were reading humorous jokes relative to non-humorous controls which differed only in the last sentence. It was found that jokes were not only rated as more humorous, recalled better, and more importantly read faster in the last sentence (the punchline). Since the participants were required to rate the funniness of the materials during the experiment, these authors attributed the longer reading times to the re-examination of possible humorous contents that may have been missed during the initial reading. Such a facilitative effect was also supported by Ferstl et al.’s (2017) eye-tracking study. To disentangle the cognitive and affective process involved in joke comprehension, Ferstl and colleagues compared jokes with texts involving revision of the situation model without being funny. According to their results, jokes were not only read faster than the revision texts but also induced fewer regressions back to the previous context. Therefore, these authors attributed these advantages to the positive affect elicited by jokes.

Besides the facilitative effect reported in jokes, Zheng and Wang (2022) also observed a similar effect using another important type of verbal humor, homophone puns. In this study, the participants were required to rate the funniness of three types of sentences: homophone puns (e.g., 陈氏男科医院, 您的男题我们解决。*Chen’s Andrology Hospital, your male problems we solve*); unfunny congruent controls (e.g., 陈氏男科医院, 您的难题我们解决。*Chen’s Andrology Hospital, your difficult problems we solve*) and unfunny incongruent controls (e.g., 陈氏牙科医院, 您的男题我们解。*Chen’s Dental Hospital, your male problems we solve*). As they expected, these authors observed longer fixation times on the homophones in the pun condition (e.g., 男题, *male problem*) than in the congruent controls (e.g., 难题, *difficult problem*). However, since the homophones used in the congruent controls were more salient than those in the pun condition, it was difficult to decide whether the difficulty had arisen from the salience difference or from the extra effort needed for extra cognitive processes (e.g., frame shifting). Nevertheless, a reverse pattern was found regarding sentence reading times. Specifically, the authors found that the participants read the homophone puns faster than the two control conditions before moving on to the funniness rating task. Zheng and Wang (2022) argued that this finding could reflect a humor facilitation effect where the readers felt more confident to move on after they had gotten the pun.

Taken together, disputes still exist in the literature concerning whether humor comprehension can exert a facilitative effect on online cognitive processes. This inconsistency, firstly, could be partially due to the different materials used in the experiments. For example, the experimental materials used by Coulson et al. (2006) consisted of only one sentence, the final word of which could either turn the sentence into a joke or unfunny control (e.g., *She read so much about the bad effects of smoking; she decided to give up the reading/habit*.). While the jokes used by Mitchell et al. (2010) and Ferstl et al. (2017) were composed of several sentences. More importantly, the prolonged reading times for jokes reported by Coulson et al. (2006) were only reading times of these sentence-final words. In contrast, evidence supporting the humor facilitation effect was mainly based on reading times at the sentence level, usually those of the punchlines. Indeed, Zheng and Wang (2022) found prolonged reading times for the homophones in homophone puns, partly supporting the humor processing disadvantage predicted by the Space Structuring Model (Coulson et al., 2006); however, total reading times of the pun sentences were significantly shorter than their congruent controls differing only in the homophones, suggesting a humor facilitative effect could have occurred at a later stage. In addition, experimental tasks could have contributed to the above-mentioned discrepancies as well. So far, evidence supporting the humor facilitation effect is mainly from studies using funniness rating tasks (e.g., Mitchell et al., 2010; Zheng and Wang, 2022). As a result, more empirical data are needed to distinguish whether the facilitative effect is resulted from the positive feedback from humor *per se* or due to some specific strategies that readers formed during the funniness rating task, or both.

Answers to these questions may not only shed more light on the cognitive processing of humor but also provide new insights into the interaction between bodily status and cognition. Previous research on embodied cognition has demonstrated that certain bodily postures or status could influence how people perceive verbal humor (Strack et al., 1988; Kaspar et al., 2016). In a recent study exploring the humor-body association, Xu et al. (2022) abstracted the most frequent metaphors concerning humor and laughter based on a large-scale corpus investigation. They then primed the participants with some of these metaphors by asking them to perform related actions (e.g., “holding one’s belly while bending forward and backward repeatedly,” corresponding to the Chinese idiom “捧腹大笑”) before they rated the materials containing jokes. Their results showed that the participants who were primed with these embodied humor metaphors reported higher funniness scores than the participants in the control group who were primed with non-metaphor actions. This finding provides more evidence that bodily posture or status can exert a feed-forward influence on how we perceive humor. However, it is still unclear whether particular bodily experiences (e.g., the feeling of amusement) can provide immediate feedback that can modulate cognition. As a result, this unanswered question has also motivated the present investigation.

To sum up, the present study attempts to answer the following research question: whether the facilitative effect in reading humorous texts reported in previous studies is due to positive feedback from the affect system or merely derived from task-related strategies? Since most of the studies that reported such a facilitative effect have employed a funniness rating task, it was possible that the task requirements rather than enhanced cognitive efforts have led participants to spend more time reading the unfunny text in case they would miss some potential funny contents, hence the shorter reading times for their humorous counterparts (e.g., Mitchell et al., 2010). To better compare with some of the previous research, the participants read one-line sentences adopted from Zheng and Wang’s (2022) study while their eye movements were recorded. They needed to rate the comprehensibility of each sentence, which could be a homophone pun, an unfunny congruent control, or an unfunny incongruent control. If the participants would still exhibit a facilitative effect in reading the homophone puns, it would be more reasonable to claim that such an advantage may result from the humor experience itself rather than from task-based strategies. Besides, the participants were also asked to rate the same set of sentences after the eye-tracking experiment. If the facilitation effect for cognition was valid, it could be predicted that there should be a positive correlation between the facilitation and the amusement that readers received from humor.

## Methods

### Participants

A group of 32 native Chinese speakers (11 males and 21 females, mean age = 22.8, SD *=* 2.6) participated in the current study. The participants, recruited through the campus forum of a university in China, had normal or corrected-to-normal vision and were paid a small amount of money after the experiment. The study received approval from the research ethics board of the university.

### Materials

The experimental materials were adopted from the study of Zheng and Wang (2022) on homophone-pun comprehension, including a total of 72 one-line sentence triads falling into three conditions: homophone puns, congruent controls, and incongruent controls. The homophone puns were mostly collected from newspaper headlines, in which the less salient homophones were presented visually.^1^ The congruent controls were the same as their homophone-pun counterparts except the salient homophones were used instead. The incongruent controls were also created from the homophone puns by just replacing the critical context nouns supporting the less salient homophones with unrelated ones, matched in both lexical frequencies and stroke numbers (*p*s > 0.10). See Table 1 for an example trial of the three conditions and Table 2 for the lexical properties of the critical noun.

**Table 1:** Sample sentences and the setup for different region of interests (ROIs).

Condition	Example sentences	ROI_1_ (critical context noun)	ROI_2_ (homophone)	ROI_3_ (spill-over region)
Congruent control	陈氏男科医院, 您的难题我们解决。 (Chen’s Andrology Hospital, your difficulties we solve.)	男科 (andrology)	难题 (difficult problems)	我们解决 (we solve)
Homophone pun	陈氏男科医院, 您的男题我们解决。 (Chen’s Andrology Hospital, your male problems we solve.)	男科 (andrology)	男题 (male problems)	我们解决 (we solve)
Incongruent control	陈氏牙科医院, 您的男题我们解决。 (Chen’s Dental Hospital, your male problems we solve.)	牙科 (dental)	男题 (male problems)	我们解决 (we solve)

English translations were given in parentheses.

**Table 2:** Properties of context noun used in the three experimental conditions.

Sentence type	Mean word frequency	Mean stroke number	Semantic relatedness
Congruent control	20.54	16.93	2.35
Homophone pun	20.54	16.93	3.69
Incongruent control	22.03	17.18	1.95

“Semantic Relatedness” refers to the extent to which the critical context noun is semantically related to the presented homophone. Word frequency is measured in occurrences per million.

Readability of the experimental sentences was pre-rated by 45 students through an online questionnaire, which showed that the congruent controls were as statistically understandable (*M =* 3.81, SD *=* 0.37) as the homophone puns (*M =* 3.91, SD *=* 0.44, *p* > 0.10), while the incongruent controls (*M =* 3.44, SD *=* 0.45) were more difficult to understand than the pun sentences (*p* < 0.001).

Using a Latin square design, the 72 sentence triads were divided into three counterbalanced lists, so that each participant would only see one sentence from any particular sentence triad. In addition, a total of 48 fillers (36 unfunny ones) from the same source as the homophone puns were added to further lower the participants’ expectation of a humorous text. Therefore, the participants read 120 sentences during the eye-tracking experiment, including 24 homophone puns, 24 congruent controls, 24 incongruent controls, and 48 fillers. Among these sentences, humorous sentences accounted for about 30%.

### Apparatus

Eye movement data were collected from the right eye with the SR Research Eyelink 1000 plus system, at a sampling rate of 1,000 Hz. The text was displayed on a 19-inch monitor (Dell P1917S) with a refresh rate of 75 Hz and a screen resolution of 1024*768 pixels. A chin rest with forehead support was used for all participants to maximize the tracking accuracy throughout the experiment.

### Procedures

At the beginning of the eye-tracking experiment, the participants were briefly introduced to how the eye tracker works and were instructed to rate the readability of each sentence they read. The participants were seated 72 cm from the monitor. A three-point horizontal calibration was implemented and revalidation was carried out when necessary. The average validation error was within 0.5° of visual angle.

Each trial started with a cross sign displayed on the left side of the screen. Once a stable fixation at the cross sign was detected for 500 ms, a sentence would appear with its first character replacing the cross. Otherwise, the calibration procedure would be initiated in 5 s. The participants read in a normal manner and needed to press the space bar on the keyboard after they finished reading the sentence. Then, they rated the readability of the sentences on a 5-point Likert scale by mouse-clicking the corresponding number on the screen. To avoid superficial ratings, a yes/no comprehension question would follow in one-quarter of the sentences.

**Figure 1:**
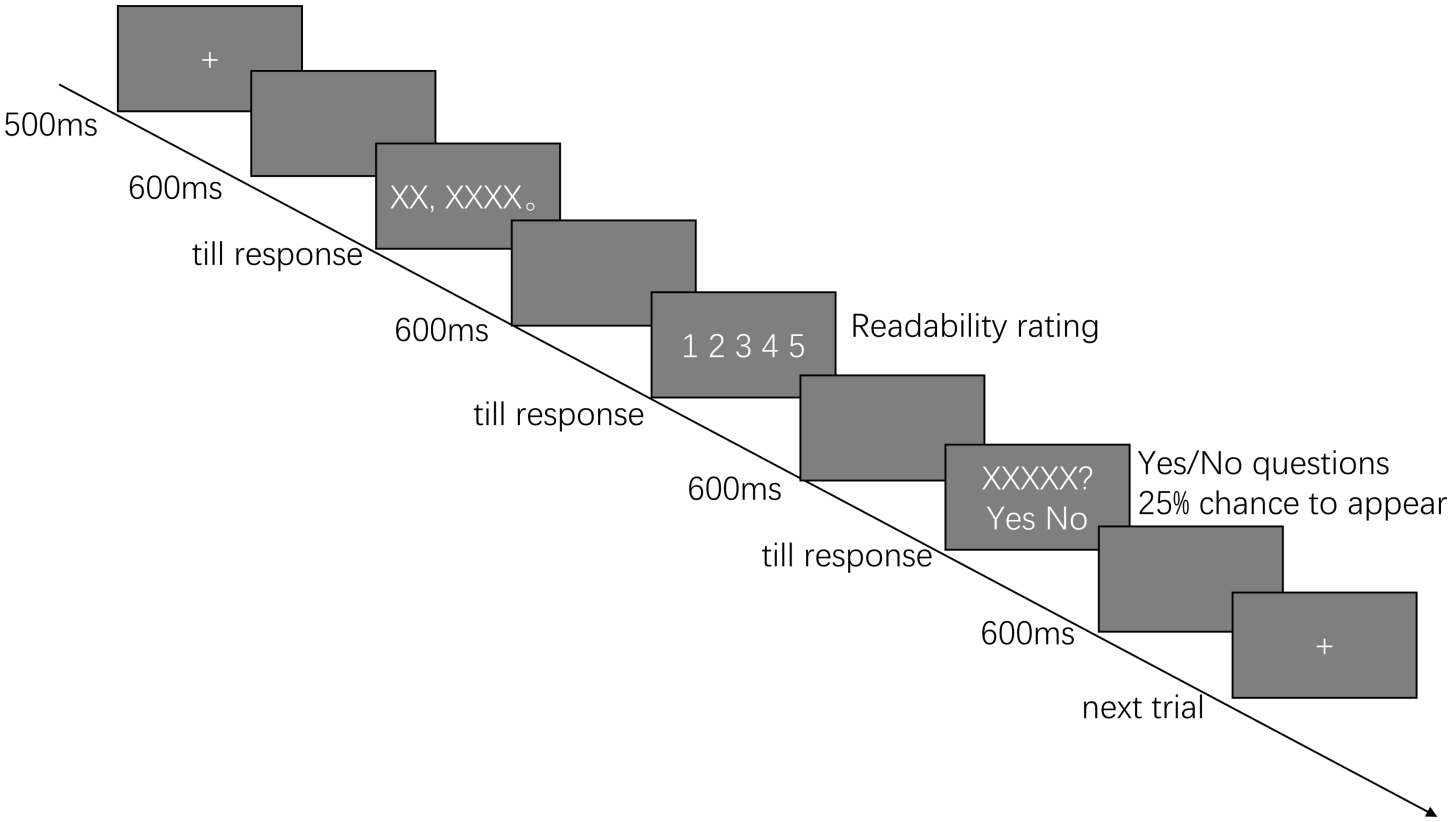
An illustration of the procedures of the eye-tracking experiment.

Each participant finished six practice trials to familiarize themselves with the experimental procedures and could try again if needed. The 120 experimental trials were pseudo-randomly assigned to four 30-trial blocks so that sentences from the same condition would not appear three times consecutively. The participants took a short break between each block. The whole eye-tracking experiment lasted for approximately 40 minutes. The procedures of the eye-tracking experiment are illustrated in Figure 1.

After the eye-tracking experiment, the participants took a rest for about 5 minutes and then were given a paper questionnaire to rate the funniness of the sentences they had previously read on a 5-point Likert scale.

## Results

The mean accuracy from the comprehension questions was 89.7% (SD *=* 6%), suggesting that the participants, in general, had understood the experimental sentences. Data from one participant was dropped from further data analysis due to low comprehension accuracy (66.7%). Moreover, trials indicating insufficient cognitive processing were eliminated (affecting around 2.6% of the data), including trials where less than two (out of the five) interest areas were visited, trials with more than two blinks, and trials with a total reading time less than 1,000 ms or greater than 2.5 standard deviations from their condition mean.

All statistical analyses were conducted using R (version 3.6.3, R Development Core Team, 2019). For analyses using continuous data (e.g., reading times) as the dependent variables, linear mixed-effect models (LMMs) were constructed using the *lmer* function from the lme4 package (Bates et al., 2015). Compared with traditional analyses, such as *t*-test or ANOVA, this method can disentangle fixed effects (experimental effect) from random effects (e.g., participant and item variations). In addition, the LMMs can produce robust results even when the sphericity and homoscedasticity assumptions are not met (Cunnings, 2012).

Following the recommendation of Barr et al. (2013), we started the model with a maximum random effect structure, including a random intercept and slope for each participant and trial item. In cases where the model failed to converge or indicated a singularity issue, the by-item random slop was dropped, followed by the by-participant random slop if necessary. Model comparisons were conducted using the ANOVA function to select better models with lower AIC values. Besides, log-transformations were used on the time-related measures to better meet the assumption of normal distribution of the residuals. Considering the small number of fixed and random effects and the large number of observations estimated, effects with a *t* or *z* value greater than 2 were treated as significant.

### Sentence-level analysis

**Figure 2:**
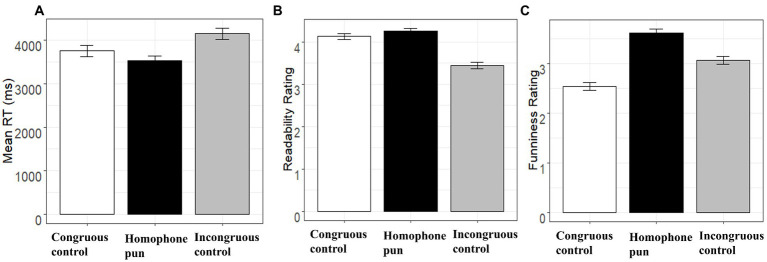
Summary statistics at the sentence level. Sentence reading time, readability rating, and funniness rating for the three sentence conditions are shown in panels **(A–C)**, respectively.

Sentence-level analyses were conducted on the reading times, readability rating scores, and funniness rating scores of the three different conditions. See Figure 2 for a summary of each measure.

#### Sentence reading time

Sentence reading time (*RT*) was defined as the duration from the presentation of a sentence to the moment the participants pressed the space bar on the keyboard. The average sentence reading times of the three sentence types can be seen in Figure 2 (Panel A). LMMs were built to analyze the relationship between the sentence *RT* and the sentence *Type* controlling the factor of sentence *Length*. We started the model with a maximum random effect structure: lmer(log10(RT) ~ Type + Length + (1 + Type|Subject) + (1 + Type|Item), data = Data).

According to the analysis, the pun sentences (*M =* 3,525 ms, SD *=* 1,481 ms, *t =* −2.56) were read significantly faster than both the congruent controls (*M =* 3,754 ms, SD *=* 1786 ms), and the incongruent controls (*M =* 4,147 ms, SD *=* 1745 ms, *t =* 9.75). This finding shows that even when the participants were required to read for comprehension, the homophone puns were still read faster than the congruent controls. As a result, this pattern is consistent with that reported by Zheng and Wang (2022), where the participants were required to focus on the affective side of the same set of materials by conducting a funniness rating task.

#### Readability and funniness ratings

The average readability scores of the three conditions are shown in Figure 2 (Panel B). Consistent with the readability ratings acquired through the online questionnaire, the homophone puns (*M =* 4.27, SD *=* 0.45) were rated as understandable as the congruent control sentences (*M =* 4.14, SD *=* 0.45, *p* > 0.10), indicating that the observed difference in sentence reading time was unlikely due to readability differences in the two sentence conditions. On the other hand, incongruent controls (*M =* 3.47, SD *=* 0.64, *p* < 0.01) were rated more difficult to understand than the other two conditions. Pairwise comparisons with Bonferroni correction showed that the homophone puns (*M =* 3.63, SD *=* 0.62) were rated significantly funnier than both the congruent controls (*M =* 2.53, SD *=* 0.67, *p* < 0.01) and the incongruous controls (*M =* 3.08, SD *=* 0.54, *p* < 0.01).

The average funniness rating scores of the three conditions are illustrated in Figure 2 (Panel C). To examine whether the feeling of mirth can have a facilitative effect on cognitive decision latencies, we also analyzed the relationship between the *RT* and the funniness rating scores (*Fun*) controlling the factor of sentence *Length* with the following formula: lmer(log10(RT) ~ Fun + Length + (1 + Type|Subject) + (1 + Type|Item), data = Data). It was found that the sentence reading time was negatively related to the funniness rating scores (*β* = −0.01, SE = 0.00, *t* = −2.35), suggesting the funnier the participants rated the sentence the faster they would finish the rating task.

### Interest-area analysis

Prior to the analyses, fixations shorter than 80 ms or longer than 1,200 ms were excluded from further analysis (affecting around 4.6% of data). Three regions of interest (ROIs) were set up for eye movement analyses, including the critical context word area (ROI_1_), the homophone area (ROI_2_), and the spillover area (ROI_3_). The ROI_1_ was set up to examine the context effect, the ROI_2_ to investigate the processing of the homophones, and the ROI_3_ to capture possible spillover effects resulted from the homophones. See also Table 1 for an illustration of the setup for different ROIs.

The following eye-movement measures were analyzed. Gaze duration (GD, also called *first-pass time*) is the total duration of all fixations from entering a region until existing either to the right or to the left. Total duration (TD, also called *dwell time*) is the summed duration of all fixations in the region. Regression Path Duration (RPD) is the total duration of all fixations that occur from the first fixation on a region until the target region is exited to the right. Regression-In proportion (Reg_In) and Regression-Out proportion (Reg_Out) is defined as the probability that readers regress into or out of a certain region, respectively.

Among the fixation-based measures, GD is sensitive to early lexical processing, and TD can reflect both the early lexical processing and the semantic integration processes (Rayner et al., 2004). The regression-based measures, RPD, Reg_In, and Reg_Out, can index extra cognitive effort that readers have to make, especially when they experience difficulty in integrating the current input and have to search for more clues in previous regions.

Mean fixation times and the standard error for the eye movement measures are shown in Table 3. Eye movement measures were defined as dependent variables and subjected to a series of linear mixed-effect models using the homophone pun condition as the baseline for comparisons.

**Table 3:** Fixation data of different regions of interest (ROIs) in the three experimental conditions.

	ROI_1_ (critical context noun)			ROI_2_ (homophone)			ROI_3_ (spill-over region)		
	CC	HP	IC	CC	HP	IC	CC	HP	IC
GD (ms)	271(6)	260(6)	272(6)	324(8)	390(9)	397(9)	373(11)	382(11)	408(13)
TD (ms)	431(12)	392(11)	480(14)	545(14)	633(14)	753(18)	642(23)	643(20)	724(23)
RPD (ms)	726(30)	729(28)	709(26)	1,136(55)	1,124(55)	1,428(62)	1,672(56)	1,630(50)	1991(60)
Reg_In (%)	36.9(2)	29.5(2)	36.0(2)	27.1(2)	32.7(2)	35.5(2)	NA	NA	NA
Reg_Out (%)	21.3(2)	20.5(2)	20.4(2)	27.3(2)	22.8(2)	25.6(2)	82.8(2)	77.3(2)	84.4(2)

CC, congruent control; HP, homophone pun; IC, incongruent control. Standard errors are reported in parentheses.

#### The critical context noun region (ROI_1_)

For ROI_1_, early measures revealed no significant difference between the congruent control and the pun sentences in GD (*β =* 0.01, SE *=* 0.01, *t =* 1.01), which was as expected since the same critical context word was used. However, the critical context words were read significantly slower in the congruent controls than in the homophone puns in TD (*β =* 0.04, SE *=* 0.01, *t =* 2.77). Regression-In data showed that the participants were more likely to regress to the context noun region in the congruent control condition (*β =* 0.38, SE *=* 0.13, *z* = 2.83, *p* < 0.01).

Gaze-duration analyses revealed no significant difference between the pun sentences and the incongruent controls in ROI_1_ (*β =* 0.01, SE *=* 0.01, *t =* 0.98), indicating that the lexical properties of the critical context nouns were well-matched. As in the congruent control condition, the participants also spent longer times in ROI_1_ in the incongruent control condition in terms of TD (*β =* 0.07, SE *=* 0.01, *t =* 4.93) and were more likely to regress into this region (*β =* 0.33, SE *=* 0.13, *z* = 2.46, *p* < 0.05).

#### The homophone region (ROI_2_)

For the homophone region, fixation-based measures revealed that the less salient homophone in the homophone pun condition was read significantly slower than its salient homophone mate in the congruent condition (GD, *β =* −0.07, SE *=* 0.01, *t =* −6.09; TD, *β =* −0.08, SE *=* 0.01, *t =* −6.18). However, regression-based measures showed another pattern: the participants were less likely to reexamine this region in the former condition (Reg_In, *β =* −0.33, SE *=* 0.13, *t =* −2.51, *p* < 0.05) and there was no significant difference between the two conditions in terms of RPD (*β =* 0.00, SE *=* 0.03, *t =* 0.06).

Fixation analyses showed that the less salient homophone in homophone puns was read as fast as in the incongruent condition in terms of GD (*β =* 0.01, SE *=* 0.01, *t =* 0.73), but faster in terms of TD (*β =* 0.07, SE *=* 0.01, *t =* 5.25), indicating that the homophones were easier to be integrated in the homophone-pun condition. This pattern was further supported by the longer RPD in the incongruent condition (*β =* 0.10, SE *=* 0.03, *t =* 3.95).

#### The spill-over region (ROI_3_)

In ROI_3_, analyses suggested no difference between the congruent control sentences and the homophone puns in GD (*β =* −0.01, SE *=* 0.01, *t =* −0.60) and in TD (*β =* −0.02, SE *=* 0.02, *t =* −1.06) and in RPD (*β =* 0.00, SE *=* 0.02, *t =* 0.27). However, the participants regressed significantly more in the congruent control condition (*β =* 0.39, SE *=* 0.17, *z =* 2.33, *p* < 0.05), consistent with the finding that the participants were more likely to re-examine ROI_1_ in this condition.

Analyses of gaze duration show no significant difference between the pun sentences and the incongruent sentences (*β =* 0.02, SE *=* 0.01, *t =* 1.09). In contrast, analyses on other measures revealed significant differences in TD (*β =* 0.04, SE *=* 0.02, *t =* 2.98), RPD (*β =* 0.09, SE *=* 0.02, *t =* 5.81), and Reg_Out (*β =* 0.50, SE *=* 0.17, *z* = 2.96, *p <* 0.05), suggesting it was more difficult to make sense of the incongruent sentences.

## Discussion

The current eye-tracking study has investigated whether humor experience (i.e., the feeling of amusement or mirth) can give immediate feedback to the brain and facilitate ongoing cognitive tasks. In the experiment, the participants read homophone-pun sentences and two comparison controls (congruous and incongruous). It is found that though the salient homophones in the congruent control sentences were read significantly faster than their less salient homophone mates in the homophone puns, this lexical-level advantage was overridden when regression-based measures were examined. Specifically, the participants were more likely to make regressions from both the homophone region and the following sentential-final region in the congruous control condition. In addition, sentence-level analyses have exhibited a negative correlation between sentence reading time and the funniness rating score. Since the participants were required to rate the comprehensibility instead of the funniness of the sentences during the eye-tracking experiment, it was unlikely that the participants had strategically regressed more in the unfunny control conditions to double-check for potential humorous contents. Instead, we argue that the positive feeling of mirth when getting the pun facilitated the cognitive processes of the readability rating task, hence the fewer regressions and faster sentence reading times.

### Facilitative effect in reading humorous texts

The facilitative effect in reading puns observed in the present study is consistent with previous research reporting a similar effect in reading jokes (Mitchell et al., 2010; Ferstl et al., 2017). However, unlike previous studies where the participants were usually required to rate the funniness of the materials, we required the participants to rate the readability of the sentences. Compared with a funniness rating task, this task can lead the participants to focus more on the cognitive aspects of the reading materials instead of the affective ones. Indeed, Mitchell et al. (2010) contributed the facilitative effect of jokes, at least partly, to the funniness rating task, which could have led the participants to regress more to recheck humorous contents that could have been missed. As a result, it is more conclusive for the current study to claim that reading humorous materials can facilitate online cognitive processing.

The shorter reading times for pun sentences than both the congruent and incongruent controls lend further support to Ferstl et al.’s (2017) explanation. According to these authors, such a facilitative effect in reading humorous materials (e.g., jokes) could be attributed to the instantaneous feedback about the correctness of the interpretation. Although homophone puns are used in the current study instead of jokes, puns are also an important type of verbal humor, which has been widely used in the research of humor (e.g., Goel and Dolan, 2001). As a result, we believe that it is the same mechanism that underlies the facilitative effects in both studies. In addition to reading times, Ferstl et al. (2017) also found that the regression rate from the punchline to the context region was negatively correlated with the funniness rating score in the jokes. And in the current study, we have found a negative correlation between the sentence reading time and the funniness rating score. Although the funniness rating scores were acquired after the participants finished the eye-tracking experiment, this general trend can also support this cognitive feedback account.

The faster sentence reading time for humorous materials (i.e., homophone puns), however, does not contrast with the study of Coulson et al. (2006). Ferstl et al. (2017) also compared their results with this earlier study and attributed the differences to task requirements and experimental materials. Specifically, their joke materials were dialogs consisting of several sentences, and the participants were required to finish either a funniness rating task or revision judgment, while in Coulson et al.’s (2006) study the participants only read one-line jokes and answered comprehension questions. Of course, these differences could account for some of the variations in the reading times. However, the total reading times reported in Coulson et al.’s (2006) study were the total reading times of the sentence-final words, which should not be compared directly with the sentence reading times reported in Ferstl et al.’s (2017) study. Comparatively speaking, the materials and tasks used in the current study are more comparable to those of Coulson et al. (2006). In the present study, we also used one-line sentences which can either turn into a homophone pun or a non-humorous control by the critical homophone. And the readability rating task was also supposed to focus the participants’ attention on the cognitive aspects of the text.

Indeed, the longer fixations (GD and TD) at the critical homophone regions for the homophone puns compared with the congruent controls are consistent with the findings of Coulson et al. (2006), supportive of their Space Structure Model. According to this theory, understanding homophone puns involves a frame-shifting process (retrieving a new frame from the long-term memory), which is cognitively more effortful and increases the reading times for words that trigger this process (namely, the less salient homophones). Of course, the difference in GD can at least partially be resulted from the lexical properties of the homophones since less salient homophones are used in the homophone pun condition. However, the differences in TD indicate more difficulty in integrating the less salient homophones into the context, presumably indicating the extra effort involved in the process of frame-shifting. In addition, this difficulty seemed quite local and we did not observe a significant spill-over effect in the following region. It is worth noting that this local (lexical) processing difficulty in reading homophone puns is not necessarily in conflict with the global (sentential) advantage of this type of humorous material. On the contrary, this reverse pattern highlighted the positive feedback of humor was derived after the participants had gotten the pun.

In addition, the significantly longer fixations for both the ROI_2_ and ROI_3_ in the incongruent condition relative to the pun condition indicate that the incongruity caused by the less salient homophone was detected. Note that the incongruent condition was created from the homophone pun condition by replacing the critical context noun with an unrelated noun. Although this manipulation made the less salient homophone difficult to process, the participants could still make some sense out of the sentence with the meaning of the unpresented salient homophones, as in the homophone pun condition. According to the Incongruity-Resolution Model, the resolution of incongruity should elicit the feeling of amusement in humorous texts (Suls, 1972). However, funniness rating scores for the incongruous condition (*M* = 3.08) indicate more uncertainty rather than amusement. As a result, this discrepancy indicates the other important prerequisite of humor experience, namely a nonserious playful mindset (Wyer and Collins, 1992). In the homophone pun condition, the participants were more likely to treat the less salient homophones as “a play on words” in the pun condition while as “spelling mistakes” in the incongruent condition. In fact, some of the participants did express their doubt about potential misspellings in the incongruent condition after finishing the experiment.

### Interaction between the body and cognition

The current findings can shed new light on the embodied cognition approach to humor studies. One of the central claims of the embodied cognition theories is that our knowledge or cognition is derived from our interaction with the outside world through our bodied experience, and therefore is fundamentally anchored in multiple bodily ways such as simulations, situated actions, and bodily states (Barsalou, 2008). An increasing literature has accumulated supporting this embodied view of language (See Barsalou, 2010 for a review). One of the most extensively used paradigms in this line of research is to prime participants with certain sensorimotor experiences that are associated with abstract concepts and then evaluate the influence they may have on the ensuing processing of the corresponding concepts. For example, Kaspar et al. (2016) asked some of their participants to pull a manikin leg or yank a chain before they added captions for cartoons. According to the funniness ratings of independent judges, these participants generated funnier captions than the control group who received no such action primes, suggesting that these bodied actions (connected with metaphorical expressions, i.e., *pulling one’s leg* and *yank one’s chain*) may have a “feed-forward” impact on the ongoing language tasks. In contrast, the participants in the current study were faster in finishing rating the more humorous homophone-pun sentences without any bodily prime before the task, indicating the bodily state of feeling mirth can also exert a “feed-backward” influence on ongoing cognitive tasks. As a result, the current study lends more support to the embodied cognition view of language processing.

The interaction between cognition and affect observed in the current study can be better explained by models that implicitly distinguish the cognitive and affective aspects of verbal humor. Chan et al. (2013), for example, proposed a three-staged model of verbal humor processing based on their fMRI study, including incongruity detection, incongruity resolution, and humor elaboration (See also Wyer and Collins, 1992). According to their findings, these three stages have their specific neural signatures in the brain: the incongruity-detection stage is characterized by greater activation in the right middle temporal gyrus and right medial frontal gyrus, the incongruity-resolution stage is associated with greater activation in the left superior frontal gyrus and left inferior parietal lobule, and the feeling of mirth activates rewarding areas such as the subcortical bilateral amygdalae and bilateral parahippocampal gyri. These findings have provided convincing evidence that the cognitive and affective aspects associated with humor are underpinned by discrete neural networks. As a result, this cognitive-affective model can delineate a more complete picture of humor processing than the classic cognitive-perceptual models, such as the incongruity-resolution model (Suls, 1972).

One limitation of the current study needs to be noted. Namely, the funniness rating scores obtained in this study could be affected by the experimental procedures. Since our first concern is to disentangle the positive feedback triggered by the humor experience from the potential strategic influence formed by the funniness rating task, the funniness rating scores were collected after the eye-tracking experiment. Although the general patterns in the ratings of the three sentence types were consistent with that of Zheng and Wang’s (2022) study, some of these scores could suffer from the fatigue or practice effect.

## Conclusion

In conclusion, the current study provides new evidence that the feeling of mirth that we experience from reading humorous texts can send instantaneous feedback to our cognitive system and facilitate ongoing cognitive tasks. This finding is consistent with the embodied cognition view of language, which emphasizes the essential role of bodily experience in shaping our cognition. In addition, this interaction between body and cognition also suggests humor theories should incorporate both the cognitive and affective components to better account for our humor experience.

## Data availability statement

The raw data supporting the conclusions of this article will be made available by the authors, without undue reservation.

## Ethics statement

The studies involving human participants were reviewed and approved by Research Ethics Board of Zhejiang University. The patients/participants provided their written informed consent to participate in this study.

## Author contributions

WZ and XW conceived and designed the experiments and revised the manuscript. WZ performed the experiments and analyzed the data and wrote the manuscript. All authors contributed to the article and approved the submitted version.

## Funding

This study is supported by the Major Project of National Social Science Foundation of China (14ZDB155).

## Conflict of interest

The authors declare that the research was conducted in the absence of any commercial or financial relationships that could be construed as a potential conflict of interest.

## Publisher’s note

All claims expressed in this article are solely those of the authors and do not necessarily represent those of their affiliated organizations, or those of the publisher, the editors and the reviewers. Any product that may be evaluated in this article, or claim that may be made by its manufacturer, is not guaranteed or endorsed by the publisher.

## Footnotes

^1^A group of 30 judges was invited to write down the first word they can think of according to the homophonic sounds (expressed in Chinese *pingyin*). Homophones written down by more than 90% of the judges were defined as the salient homophones; the other homophonic alternatives were regarded as the less salient ones (Giora, 1997).
